# Diabetes Mellitus Is a Possible Risk Factor for the Development of Trochanteric Bursitis—A Large-Scale Population-Based Study

**DOI:** 10.3390/jcm12196174

**Published:** 2023-09-24

**Authors:** Assaf Kadar, Ron Itzikovitch, Yaniv Warschawski, Samuel Morgan, Shai Shemesh

**Affiliations:** 1 Roth|McFarlane Hand and Upper Limb Centre, St. Joseph’s Health Care London, Western University, London, ON N6A 4V2, Canada; assaf.kadar@sjhc.london.on.ca; 2 Independent Researcher, Kfar Saba 4424309, Israel; ronitzik1@gmail.com; 3 Faculty of Medicine, Tel-Aviv University, Tel-Aviv 4424309, Israel; yanivarsh@gmail.com (Y.W.); samuelmorgan@mail.tau.ac.il (S.M.); 4 Division of Orthopaedic Surgery, Tel Aviv Medical Center, Tel-Aviv 6423906, Israel; 5 Department of Orthopaedic Surgery, Samson Assuta Ashdod University Hospital, 7 Ha’Refua Street, Ashdod 7747629, Israel; 6 Faculty of Health Sciences, Ben Gurion University, Beer-Sheva 8410501, Israel

**Keywords:** trochanteric bursitis, greater trochanteric pain syndrome, diabetes mellitus, gender

## Abstract

Background: Trochanteric Bursitis (TB) is a common reason to seek primary care, previously shown to be associated with female gender and obesity. Diabetes mellitus (DM) has several musculoskeletal manifestations, but was never found to be associated with TB. Purpose: To explore the association between DM and TB, based on a large database. The secondary aim was to explore the influence of gender and insulin usage on the occurrence of TB. Study design: cross-sectional study. Methods: A population-based cohort consisting of 60,610 patients (55,428 without DM and 5182 with DM), of whom 5418 were diagnosed with TB. A logistic regression model was applied to estimate propensity scores. Results: The odds of individuals with DM being diagnosed with TB were 55.8% higher compared to the odds of patients without DM (OR: 1.558, 95% CI: [1.429, 1.70], *p* < 0.0001). We found that insulin users had a lower risk of TB than patients not using insulin (log-rank *p* < 0.0001). Females are 3.3 times more likely to have TB than males (RR: 3.337, 95% CI: [3.115, 3.584], *p* < 0.0001). Conclusions: DM is a risk factor for developing TB. Insulin had a protective effect against TB, suggesting that better glycemic control might prevent this painful infliction.

## 1. Introduction

Trochanteric bursitis (TB) is one of the most common types of bursitis, now more commonly acknowledged to be a part of the umbrella term of greater trochanteric pain syndrome (GTPS) [1,2,3,4,5,6]. GTPS encompasses a spectrum of disorders including TB, abductor tendon tears, abductor tendinopathy and external coxa saltans [5]. As the trochanteric bursa lies deep to the iliotibial band and is superficial to the gluteus medius, tendinosis of this tendon is thought to be the culprit of TB [7,8]. Yet, risk factors for developing TB are not well understood [5]. Segal et al. found that female gender, obesity and low back pain are associated with TB [6]. The higher prevalence in women could be explained by the flared pelvic rim in females that may alter the pull of the iliotibial band, hormonal effects that may cause irritation of the trochanteric bursa, or the differences in activity between men and women [6].

Diabetes mellitus is a disease known to affect many organs and systems, such as the kidney, eyes and blood vessels [9], with a higher prevalence of joint and connective-tissue diseases as compared to the general population. The proposed pathophysiologic mechanism involves the accumulation of advanced glycation end products (AGEs), with cross-linking of collagen and other macro-molecules in musculoskeletal tissues [10]. Insulin was previously shown to have an anti-inflammatory and organ-protective effect [11,12]. We hypothesized that DM could be a risk factor for developing TB, as the trochanteric bursa and adjacent tendons may theoretically be affected by hyperglycemia. Furthermore, we expect to find a higher risk of developing TB among female patients, and a lower risk of developing TB with insulin usage.

The purpose of the following study was to explore the association between diabetes and TB, based on a large database. The primary aim was to determine the odds of being diagnosed with TB for diabetic patients compared with non-diabetic individuals. The secondary aim was to determine whether the risk of developing TB over time was associated with patient gender as well as insulin use.

## 2. Materials and Methods

A population-based cohort spanning a 15-year period, from 2005 to 2020, from the Clalit healthcare services database was used for this retrospective study. Clalit health services is the largest health provider and insurer in Israel, insuring over 65% of the population [13]. Following approval of the institutional review board, we searched the Clalit health service database for patients with a diagnosis of trochanteric bursitis.

**Figure 1:**
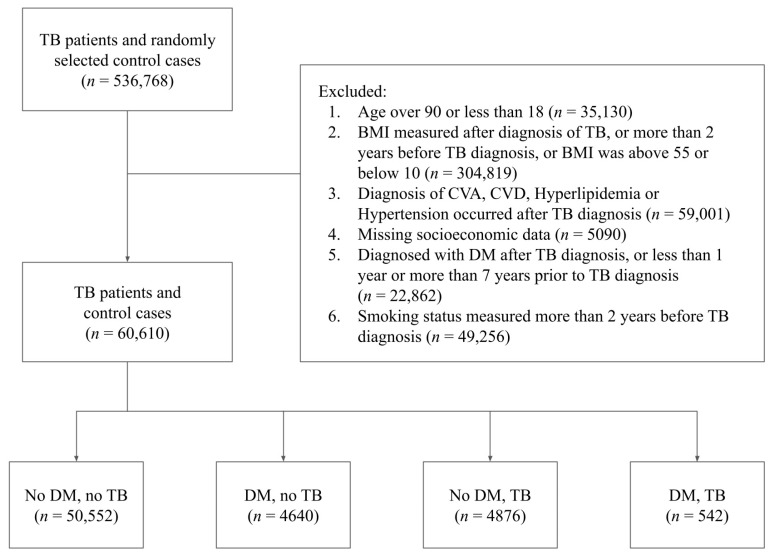
Flow chart of study participants describing the exclusion process and final study population. TB–Trochanteric Bursitis; DM–Diabetes Mellitus.

Given this initial database size of 536,768 subjects, we then proceeded to determine which of the subjects could be included in the final study population. An individual was included in the final study population if he or she met the following eligibility criteria: (1) the individual was either not diabetic or was a diabetic diagnosed with DM between 1 and 7 years before the diagnosis of TB; (2) the individual’s BMI measurements, smoking status, hyperlipidemia, hypertension, CVA, CVD and socioeconomic status were recorded prior to the diagnosis of TB; (3) the individual’s age was between 18 and 90, and the BMI was between 10 and 55; (4) the individual’s BMI measurements and smoking status were updated no more than 2 years prior to the diagnosis of TB; and (5) the individual did not have missing data regarding their socioeconomic status. As our data were queried from a larger database specifically for this study, we opted to remove from the initial study population all patients who were diagnosed with DM but went into remission, as this group of patients was not considered relevant with regards to our research question. Figure 1 describes the full data screening process through which the final study population was chosen.

Age, BMI and socioeconomic status were categorized to facilitate a more interpretable analysis, and these categorized variables were used as covariates in our model. The age variable was categorized in the following manner: age 18 to 44, age 45 to 54, age 55 to 64, age 65 to 74 and age 75 to 90. The variable BMI was split into categories as well: BMI 10 to 18.4, BMI 18.5 to 24, BMI 25 to 29, BMI 30 to 34, and BMI 35 to 55. We used a three-scale classification of high, medium and low to classify the socioeconomic status of the individuals in the study. Smoking was treated as a binary variable (Yes/No), which indicated whether the individual was considered a smoker in the two years prior to the diagnosis of TB. Past medical history included hyperlipidemia, hypertension, cerebrovascular accident (CVA) and cardiovascular disease (CVD). These variables were also included in the analysis as binary (Yes/No). Individuals who were diagnosed with any of hyperlipidemia, hypertension, CVA or CVD after the diagnosis of TB were excluded from the study.

A total of 60,610 individuals were included in the final study population (mean age: 58.13, SD: 15.3), of which 34,296 (56.59%) were females and 26,314 (43.41%) were males. A total of 5182 (8.56%) of the individuals in the cohort were diagnosed with DM and 55,428 (91.44%) were not. A total of 5418 (8.9%) of the individuals in the cohort were diagnosed with TB and 55,192 (91%) were not. Table 1 describes the baseline characteristics of the subjects in the study.

The Strengthening the Reporting of Observational Studies in Epidemiology STROBE guidelines were applied and followed (Supplementary Materials).

### Statistical Methods

We used propensity score modeling with logistic regression and inverse probability of treatment weighting to estimate the odds ratio of developing TB for patients with and without DM. In order to obtain an unbiased estimate of the average effect of DM on TB we first built a logistic regression model to estimate the propensity scores of the individuals in the study [14,15]. The propensity score model predicted the subject’s probability of having DM, given the following covariates: gender, age category, BMI category, smoking status (Yes/No), hyperlipidemia (Yes/No), hypertension (Yes/No), CVA (Yes/No), CVD (Yes/No) and socioeconomic status (low, medium and high). We then used the propensity score model to estimate the weights of the observations in the study by using inverse probability of treatment weighting (IPTW) with stabilized weights [16,17].

Evaluation of the weighting and balancing of the groups of patients with and without DM was carried out by reviewing a numerical summary (Table 2) showing the distribution of the covariates among the weighted groups along with the absolute standardized mean difference (SMD) between the groups [18,19].

**Figure 2:**
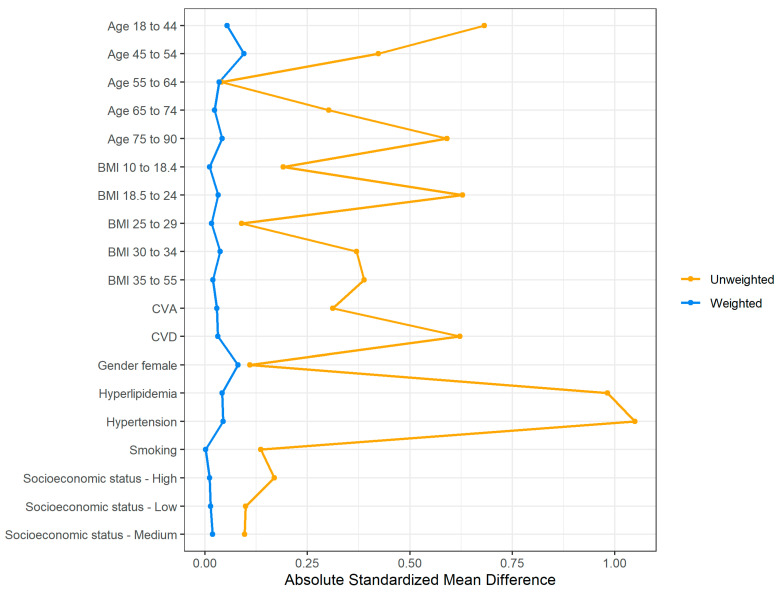
Absolute standardized mean difference between the groups of diabetic and non-diabetic patients in the unweighted vs. weighted study population.

Although the unweighted groups were not balanced in terms of the distribution of covariates, after applying the weights the absolute SMD for all covariates of interest was 0.1 or lower, which indicates that the differences in the covariates’ means between the groups are minor. A graphical summary of the absolute standardized mean difference between the weighted groups is presented in Figure 2 [16].

Once we obtained the above weights, we used them to fit two models for the weighted subjects in the study: (1) a logistic regression model which predicted TB, with DM as the only covariate. This model was used to estimate the unadjusted odds ratio; (2) a logistic regression model to estimate the association between DM and TB. The covariates used in this model were DM (Yes/No), as well as the other covariates used in the aforementioned propensity score model. This model was used to estimate the various adjusted odds ratios and adjusted relative risks. Significance level was set at 5%. Confidence intervals for the relative risks were obtained by using bootstrap percentile confidence intervals [20]. All statistical analysis was conducted in R version 4.0.2.3 (Foundation for Statistical Computing, Vienna, Austria).

## 3. Results

The proportions of patients diagnosed with TB among patients with and without DM was 13.37% vs. 9%, respectively. Considering our main hypothesis that there exists an association between DM and TB, we found that the (unadjusted) odds of individuals with DM being diagnosed with TB were 55.8% higher compared to the odds of individuals who were not diabetic (odds ratio (OR): 1.558, 95% confidence interval (CI): [1.429, 1.70], *p*-value: <0.0001). After adjusting for baseline characteristics and comorbidities, the odds of individuals with DM being diagnosed with TB were 49.1% higher compared to the odds of individuals who were not diabetic (OR: 1.491, 95% CI: [1.365, 1.629], *p*-value: <0.0001), indicating a significant and meaningful association between DM and TB. The mean of the stabilized weights (described in the Section 2) which were used to build the models was 0.997, which is very close to the ideal mean of 1, indicating a properly specified propensity score model [21].

**Figure 3:**
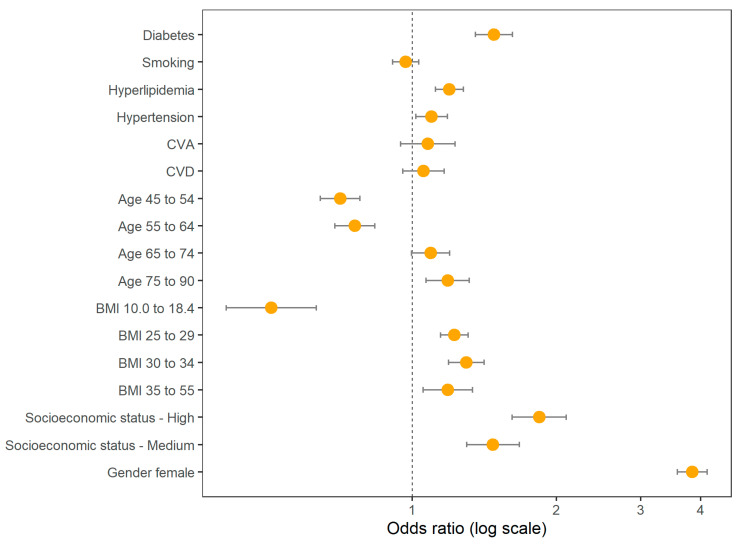
Odds ratios estimated by the fully adjusted logistic regression model. For the non-binary covariates, the relevant reference levels used to estimate the odds ratios are age 18 to 44, BMI 18.5 to 24, and low socioeconomic status.

In Table 3 we show the odds ratios for DM and the various covariates included in the adjusted model, and these are also shown graphically in Figure 3.

In order to compare the adjusted relative risk (RR) of individuals with DM vs. those who do not have DM, we considered a group of females with no comorbidities, non-smoking, normal weight (BMI 18.5 to 24), age 55–64 and medium socioeconomic status. Within this group, the adjusted risk of TB for diabetic females was 1.432 times the risk of non-diabetic females (relative risk (RR): 1.432, 95% CI: [1.156, 1.726], *p*-value: 0.005). Given a similar group of males (same age group, BMI group, etc., as in the females group) the risk of TB for diabetic males was 1.479 times the risk of non-diabetic males (RR: 1.479, 95% CI: [1.168, 1.815], *p*-value: 0.003).

**Figure 4:**
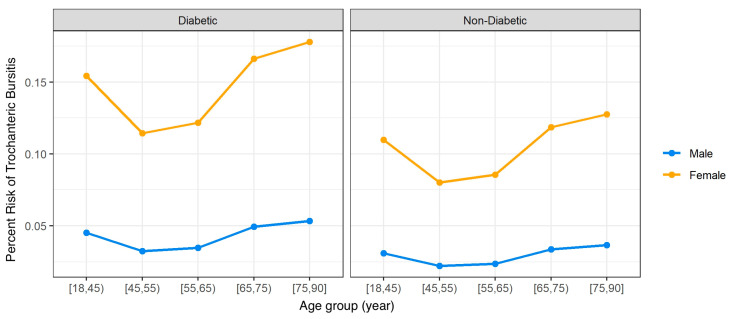
The adjusted risk of Trochanteric Bursitis for males and females, shown for subjects with and without Diabetes.

Moreover, we estimated the adjusted RR for two sub-groups of interest where gender was the differing factor. When considering subjects with DM, age 75–90, BMI 18.5–24, non-smoking with no comorbidities and medium socioeconomic status we observed that females are 3.3 times more likely to have TB than males (RR: 3.337, 95% CI: [3.115, 3.584], *p*-value: <0.0001). Further examination of the risks of males and females showed that females are at higher risk of TB than males in all age groups, as shown in Figure 4.

**Figure 5:**
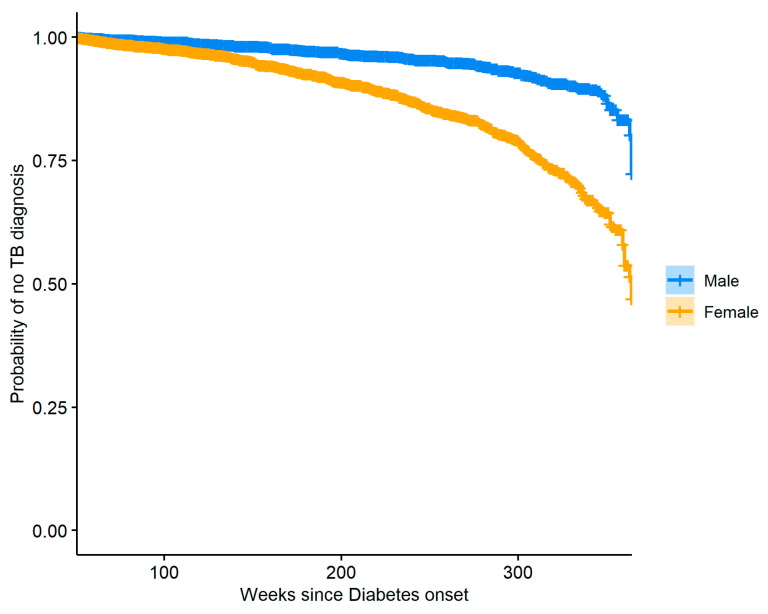
Kaplan–Meier curves stratified by gender for the group of diabetic subjects, showing the probability of no TB for diabetic females and diabetic males over time, following the diagnosis of Diabetes.

We also took interest in the group of diabetic subjects and the risk of TB over time associated with patient gender as well as insulin usage. Figure 5 shows Kaplan–Meier curves describing the probability of having TB for both male and female diabetic patients.

**Figure 6:**
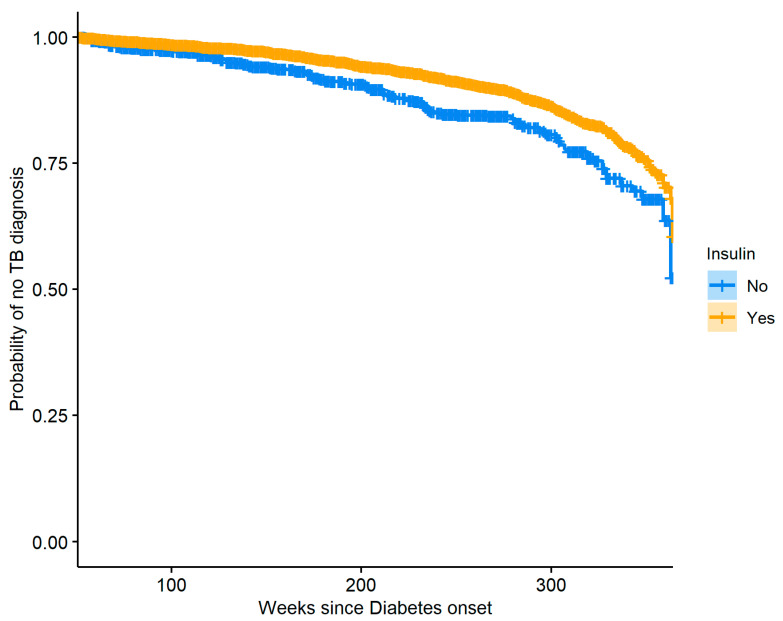
Kaplan–Meier curves stratified by insulin usage for the group of diabetic subjects, showing the probability of no TB diagnosis.

To reduce confounding, a propensity score model with gender as the outcome was used to produce stabilized weights for the observations, and these weights were then used to create the plot with balanced covariates between the weighted groups. The two curves presented in Figure 5 are significantly different (log-rank test *p*-value: <0.0001), and we see that females are generally at greater risk of TB when compared to males. The same method was used to perform a similar analysis where insulin usage was used to stratify the diabetic patients. Figure 6 shows the resulting Kaplan–Meier curves which are significantly different (log-rank *p*-value: <0.0001), indicating that over time insulin users have a lower risk of TB compared to diabetics who do not use insulin. Careful examination of the data used to create Figure 6 revealed that our dataset had a very small number of patients with DM who were diagnosed with TB in the time range of 240–290 weeks after DM onset and did not use insulin, and so our model produced a slightly biased estimate of the hazard in that region, resulting in a small plateau in the relevant section of the curve.

## 4. Discussion

This population-based study encompassing about 60,000 patients has found that there exists a strong and significant effect of diabetes mellitus on trochanteric bursitis. The odds of TB occurrence in patients with DM were 1.491 times higher than the odds of TB occurrence in patients without DM. The increased risk of TB among patients with DM was also detected when the patients were grouped by gender: females with DM were at a higher risk of developing TB compared to females without DM (relative risk: 1.432) and a similar effect was observed for males with DM, who had a higher risk of developing TB compared to males without DM (relative risk: 1.479). The risk of TB was found to be consistently higher for females when compared to males across all age groups included in the study. Another finding was that among patients with DM, those who do not use insulin are at a higher risk for TB over time compared with patients who do use insulin. While the absolute difference in proportions of patients with TB among patients with and without DM was relatively small (13.37% vs. 9%), our focus was on the significantly increased risk as expressed by the odds ratio. This approach is in accordance with standard epidemiological practices, which often prioritize relative measures of effect such as odds ratios to better capture and quantify underlying risk factors.

Diabetes is a world-wide pandemic and health problem. The disease affects many body organs and is a major cause of disability due to end organ complications, be it lower limb amputations, blindness due to retinopathy, small blood vessel disease affecting myocardial infraction, stroke, and other disabilities. The prevalence of DM is projected to reach 4.4% of the world population in 2030 [22].

Diabetes is known to have many musculoskeletal manifestations that cause disability. Such manifestations are osteoarthritis, adhesive capsulitis, carpal tunnel syndrome, trigger finger, plantar fasciitis and rotator cuff tendinitis [23]. The mechanism in which diabetes inflicts this damage is not understood. One hypothesis suggests that hyperglycemia results in collagen glycosylation, with excessive accumulation of advanced glycation end products (AGEs) in the connective tissue. A now less-soluble, and more-resistant-to-collagenase collagen accumulates in the connective tissue and alters the extracellular matrix structure and function, causing soft tissue stiffness, weakness, and hence susceptibility to tearing [24]. In another proposed mechanism, AGEs attach to their soft tissue receptors and upregulate proinflammatory mediators, causing bursitis and tendinitis [25].

Trochanteric bursitis is known to evolve from repetitive micro-trauma or prolonged compression of the bursa that lies between the greater trochanter and the skin [26]. Therefore, the authors hypothesis that neuropathy of the sensory nerves surrounding the trochanteric region is the culprit of the higher incidence of TB in DM patients. The diabetic patient may not sense early signs of compression and pain until more advanced inflammation in the bursa ensues.

The greater trochanter and surrounding tendons and soft tissue have been viewed as analogous to the greater tuberosity of the shoulder and rotator cuff tendons. As such, rotator cuff tendinopathy (RCT) is analogous to TB or greater trochanteric pain syndrome (1). While our study is the first to report an association between diabetes and TB, the association of RCT with diabetes is a well-established one. Several large cohorts from Taiwan [27], Finland [28] and France [29] have explored that association and reported rates of up to 8.8-fold increase in RCT for patients with diabetes. Ultrasound of the rotator cuff in diabetic patients demonstrated more thickness and stiffness than tendons of healthy patients [30]. The lessoned learned from RCT in diabetes, including the possibility of improving symptoms with better glycemic control [27], can certainly be applied to TB in patients with diabetes. Another important finding of the present study is that over time, insulin users had a lower risk of developing TB compared to diabetics who did not use insulin. This finding can be explained by the proposed anti-inflammatory effect of insulin. An emerging body of evidence suggests that insulin may suppress the inflammatory process, through preventing hyperglycemia and by modulating several different inflammatory molecules. For instance, insulin was demonstrated to interfere with the signal transduction of interleukin-6 (IL-6) on adipocytes, in vitro [31,32].

When performing population-based studies it is imperative to note that when comparing the exposed or treated group to the control group, they are usually not well balanced: age distribution, BMI distribution, occurrence of comorbidities and the distribution of other covariates may vary considerably between the groups. This results in a biased estimation of the effect of risk factors, e.g., if the exposed individuals are also older and more hypertensive than the unexposed individuals, the analysis method must take that into account so that these imbalances would not confound the results of the analysis. We addressed this issue by using propensity scores to balance the groups and assign weights to all individuals prior to fitting a model, thus providing us with unbiased estimates of the effect of diabetes mellitus on trochanteric bursitis.

Our study has several limitations. First, it is a retrospective study performed on a large de-identified database and based on diagnosis codes, and this naturally restricted our accessibility to the individual management and outcome of each patient. Second, we were unable to ascertain some clinically relevant information such as data regarding glycemic control, as blood sugar levels or HbA1C were not included in our analysis. Lastly, trochanteric bursitis is a clinical diagnosis with a wide differential diagnosis. However, as this is a population cohort study, we had no way of discerning the accuracy of the diagnosis made by physicians of various sub-specialties.

## 5. Conclusions

This is the first cohort study to examine the association between diabetes mellitus and trochanteric bursitis in the adult population. We found that a history of diabetes mellitus predisposes an individual to develop trochanteric bursitis, regardless of gender, age, and other comorbidities. Females are generally at greater risk of developing trochanteric bursitis when compared to males, in all age groups. Insulin use was associated with a lower risk of developing trochanteric bursitis in patients with diabetes. Our findings suggest that the peri-trochanteric area is yet another target organ for this systemic disease. Further studies will be required to elucidate the mechanism of the deleterious effect of diabetes in this area.

## Figures and Tables

**Table 1:** Baseline characteristics of the study population by subgroup with and without Diabetes Mellitus.

Variable	Without Diabetes Mellitus (*n* = 55,428)	With Diabetes Mellitus (*n* = 5128)	All Patients (*n* = 60,610)
Age group			
18–44	13,918 (25.1%)	143 (2.8%)	14,061 (23.2%)
45–54	11,784 (21.3%)	356 (6.9%)	12,140 (20.0%)
55–64	10,515 (19.0%)	902 (17.4%)	11,417 (18.8%)
65–74	11,445 (20.6%)	1759 (33.9%)	13,204 (21.8%)
75–90	7766 (14.0%)	2022 (39.0%)	9788 (16.1%)
BMI group			
10–18.4	1849 (3.3%)	35 (0.7%)	1884 (3.1%)
18.5–24	25,136 (45.3%)	908 (17.5%)	26,044 (43%)
25–29	18,929 (34.2%)	1992 (38.4%)	20,291 (34.5%)
30–34	6879 (12.4%)	1391 (26.8%)	8270 (13.6%)
35–55	2635 (4.8%)	856 (16.5%)	3491 (5.8%)
Gender			
Female	31,622 (57.1%)	2674 (51.6%)	34,296 (56.6%)
Male	23,806 (42.9%)	2508 (48.4%)	26,314 (43.4%)
Smoking			
No	36,638 (66.1%)	3089 (59.6%)	39,761 (65.6%)
Yes	18,790 (33.9%)	2093 (40.4%)	20,849 (34.4%)
Hyperlipidemia			
No	28,878 (52.1%)	1073 (20.7%)	36,730 (60.6%)
Yes	26,550 (47.9%)	4109 ((79.3%)	23,880 (39.4%)
Hypertension			
No	43,068 (77.7%)	1628 (31.4%)	44,669 (73.7%)
Yes	12,360 (22.3%)	3554 (68.6%)	15,940 (26.3%)
CVA			
No	53,394 (96.33%)	4565 (88.1%)	57,955 (95.62%)
Yes	2034 (3.67%)	617 (11.9%)	2655 (4.38%)
CVD			
No	50,905 (91.84%)	3529 (68.1%)	54,428 (89.8%)
Yes	4522 (8.16%)	1653 (31.9%)	6182 (10.2%)
Trochanteric Bursitis			
No	50,550 (91.2%)	4638 (89.5%)	55,191 (91.06%)
Yes	4878 (8.8%)	544 (10.5%)	5419 (8.94%)
Socioeconomic status			
Low	17,710 (32.0%)	1263 (24.4%)	18,973 (31.3%)
Medium	4456 (8.0%)	567 (10.9%)	5023 (8.3%)
High	33,262 (60.0%)	3352 (64.7%)	36,614 (60.4%)

BMI, Body Mass Index; CVA, Cerebrovascular accident; CVD, Cardiovascular disease.

**Table 2:** Weighted proportions of covariates within the groups of diabetic and non-diabetic individuals in the study population.

Variable	Without Diabetes Mellitus	With Diabetes Mellitus	Absolute Standardized Mean Difference
Age group			
18–44	23.87%	25.88%	0.0608
45–54	19.82%	16.27%	0.1041
55–64	18.97%	17.72%	0.0279
65–74	21.70%	22.57%	0.0197
75–90	15.82%	17.56%	0.0414
BMI group			
10–18.4	3.1%	3.22%	0.0084
18.5–24	42.92%	41.64%	0.0290
25–29	34.49%	33.61%	0.0185
30–34	13.69%	15.21%	0.0391
35–55	5.79%	6.32%	0.0174
Gender			
Female	56.6%	60.64%	-
Male	43.4%	39.36%	0.0813
Smoking			
No	65.52%	65.68%	-
Yes	34.48%	34.32%	0.0033
Hyperlipidemia			
No	60.51%	58.71%	-
Yes	39.49%	41.29%	0.0406
Hypertension			
No	73.61%	71.73%	-
Yes	26.39%	28.27%	0.0428
CVA			
No	95.58%	94.84%	-
Yes	4.42%	5.16%	0.0279
CVD			
No	89.74%	88.58%	-
Yes	10.26%	11.42%	0.0304
Trochanteric Bursitis			
No	90.09%	86.63%	-
Yes	9.01%	13.37%	0.147
Socioeconomic status			
Low	8.29%	7.87%	0.0145
Medium	60.43%	61.36%	0.0191
High	31.27%	30.77%	0.0112

BMI, Body Mass Index; CVA, Cerebrovascular accident; CVD, Cardiovascular disease.

**Table 3:** Odds ratio for TB and baseline covariates as estimated by the multivariable logistic regression model fitted to the weighted study population.

Variable	Odds Ratio	95% CI	*p*-Value
Diabetes Mellitus			
No (reference)	-	-	-
Yes	1.491	(1.365, 1.629)	<0.0001
Age group			
18–44 (reference)	-	-	-
45–54	0.702	(0.639, 0.772)	<0.0001
55–64	0.787	(0.716, 0.866)	<0.0001
65–74	1.097	(1.001, 1.203)	0.0479
75–90	1.189	(1.071, 1.319)	0.0011
BMI group			
10–18.4	0.504	(0.406, 0.626)	<0.0001
18.5–24 (reference)	-	-	-
25–29	1.219	(1.141, 1.303)	<0.0001
30–34	1.286	(1.180, 1.401)	<0.0001
35–55	1.180	(1.048, 1.328)	0.0061
Gender			
Female	3.846	(3.580, 4.131)	<0.0001
Male (reference)	-	-	-
Smoking			
No (reference)	-	-	-
Yes	0.966	(0.908, 1.028)	0.2734
Hyperlipidemia			
No (reference)	-	-	-
Yes	1.193	(1.116, 1.275)	<0.0001
Hypertension			
No (reference)	-	-	-
Yes	1.101	(1.021, 1.188)	0.0124
CVA			
No (reference)	-	-	-
Yes	1.083	(0.951, 1.234)	0.2284
CVD			
No (reference)	-	-	-
Yes	1.060	(0.960, 1.171)	0.2468
Socioeconomic status			
Low (reference)	-	-	-
Medium	1.477	(1.302, 1.676)	<0.0001
High	1.840	(1.615, 2.096)	<0.0001

BMI, Body Mass Index; CVA, Cerebrovascular accident; CVD, Cardiovascular disease.

## Data Availability

Data is available per request from the corresponding author.

## Supplementary Materials

The following supporting information can be downloaded at: https://www.mdpi.com/article/10.3390/jcm12196174/s1, Supplementary Table S1: STROBE Statement—Checklist of items that should be included in reports of cohort studies.
